# Adjustable bone plate: state of art

**DOI:** 10.3906/sag-2002-69

**Published:** 2020-11-03

**Authors:** Gazi HURİ

**Affiliations:** 1 Department of Orthopaedics and Traumatology, Hacettepe University, Ankara Turkey

**Keywords:** Fracture, bone, shortening, lengthening, adjustable, plate

## Abstract

**Background/aim:**

The success of treatment of bone fracture and defects are based on a proper contact and compression between the bone fracture fragments. Intraoperative manipulations such as bone compression or distractions are generally done in order to achieve this. However, none of the bone plates currently in routine use allow these manipulations after fixation to the bone, requiring refixation and repeated drilling, and screwing operations. Based on this shortcoming, we designed a novel adjustable bone plate (ABP) system which allows bone shortening and lengthening even after fixation to the bone surface. The aim of the paper is to clarify the unique properties of the novel bone plate.

**Materials and methods:**

In this paper, the new generation adjustable bone plate applicability, design, indication, and new characteristics in addition to conventional bone plates with review of the literature were discussed and surgical technique was demonstrated in a saw bone model.

**Results:**

This novel design allows for compression and distraction at the fracture ends post fixation as well as bone segment transfers.

**Conclusions:**

The potential of the new generation ABP plate for use in bone compression, distraction, and the segmental bone transfer is a promising invention for clinical applications in the future.

## 1. Introduction

Orthopaedic implants such as intramedullary nails, external fixators, plates, and screws are most commonly used devices in the orthopaedics practice especially in the treatment of fracture cases [1–3]. In addition to fractures, they can also be used in bone deformity corrections, bone lengthening, and reconstructions following bone resections [4,5]. In treatment of bone lengthening and bone defects, commonplace lengthening devices are external ﬁxators and intramedullary devices. However, each has its drawbacks [6–8]. In the current manuscript, a novel adjustable bone plate system designed by our research team which allows bone shortening and lengthening even after fixation to the bone surface is presented.

## 2. Materials and methods

Limb lengthening using an external fixator is associated with many problems, such as pin tract infection, pin-associated pain, scarring, and discomforts from the bulky frame [8]. Due to the high complication rates of up to 10%–20%, lengthening with these methods requires careful surveillance [8]. Consequently, the researchers came up with the new solutions and intramedullary lengthening devices have been developed for bone lengthening [9]. However, Novikov et al. presented the intramedullary lengthening devices with several complications such as abnormal pain, implant failure, joint stiffness, mechanical failure of lengthening mechanism (66%) including runaway nails, difficult-to-distract nails and nondistracting nails, delayed union, and intramedullary infection [7]. Similar report was presented by Burghardt et al. about the mechanical failure of intramedullary skeletal kinetic distractors in limb lengthening [10]. Surgeons have recently experienced success with a motorized, intramedullary nail, but paediatric use of this device can be limited due to interference with open growth plates [11]. On the other hand, conventional plates cannot be used for segmental bone transfer or bone lengthening alone with current designs and technologies [12]. Existing plates only allow up to 2 mm movement of bones in the fracture line and do not allow any further manoeuvres [13]. Furthermore, after having been applied to the bone, conventional plates do not allow adjusting the fracture line, thus a malpositioning (displacement of fracture ends) results in a further procedure to be performed which renders the surgery period 2 or 3 times longer. Accordingly, this situation results in a similar increase in the duration of anaesthesia in a patient, and risks of complication (infection, bleeding etc.) as well. Additionally, the state-of-art bone plates are of static nature, thereby requiring use of auxiliary elements such as external fixators, especially where a bone shortening or bone extension is necessary [13]. This procedure requires further medical personnel or a second fixation implant, hence, causing redundant labour loss, additional cost and needlessly, application of an additional implant to the patient, though temporarily. Based on these requirements and knowledge, we have designed (patent numbers: WO2014033088A1, US9138270B2, EP2890313B1) [14–16] a novel adjustable bone plate (ABP) “aliGn plate” (TÜBİTAK MARTEK Implantek Ltd. Gebze, Turkey) that replaces the attractive qualities of expandable intramedullary nail as well as external fixator device by minimalizing the risk of deep infection and no damage to growth plates. 

## 3. Results

### 3.1. Design and application of ABP

**Figure 1:**
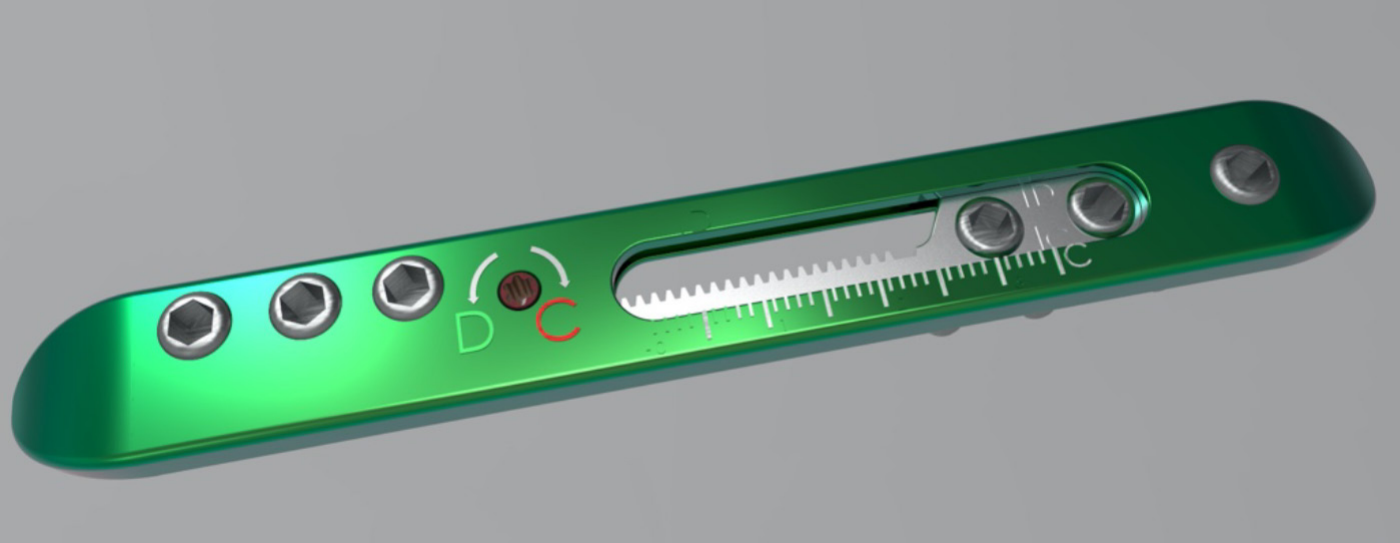
Adjustable bone plate (aliGn plate, Turkey) has static (green part) and dynamic parts (grey part).

**Figure 2:**
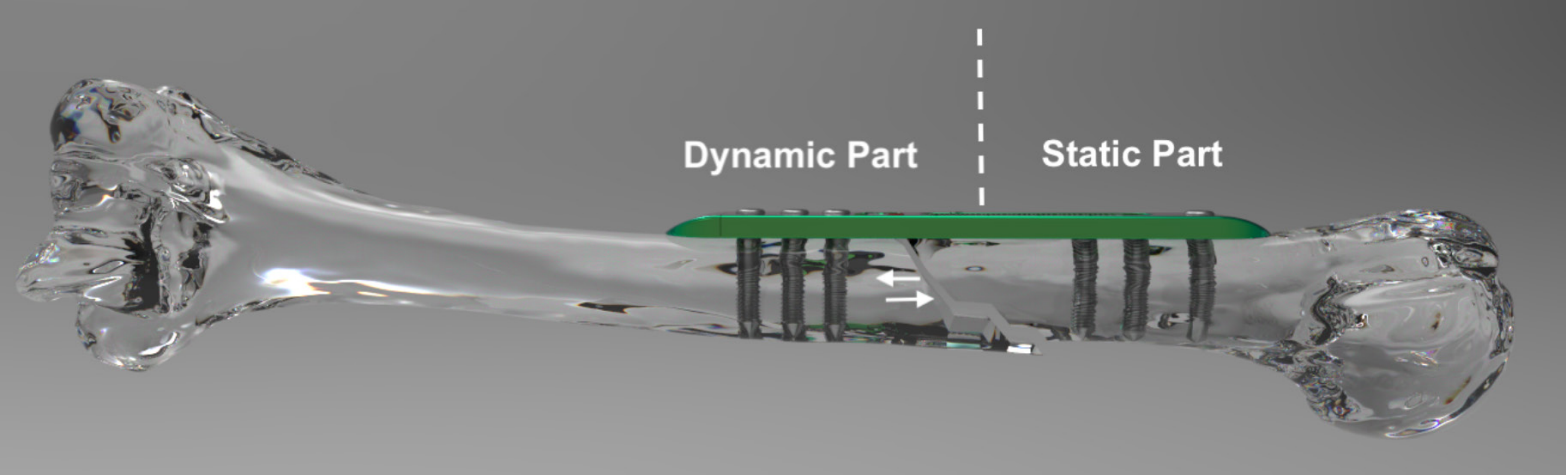
ABP (green) applied on humerus surface includes dynamic and static parts. The arrows indicate the directions of the bone transfer.

ABP is composed of static and dynamic parts (Figures 1, 2). The outer edge of the plate and the 3 holes in the ends of the ABP form the static part. Dynamic part constitutes pinion mechanism and associated screw holes. Dynamic part allows movement with the pinion mechanism. Rotating the pinion mechanism screw in “D” direction with a manual screw results in distraction at fracture line while it allows compression by rotating the pinion in “C” direction. Screw holes are similar with locking screws in LCP plate. ABP was manufactured with 3.5 mm and 4.5 mm dimension options. 

**Figure 3:**
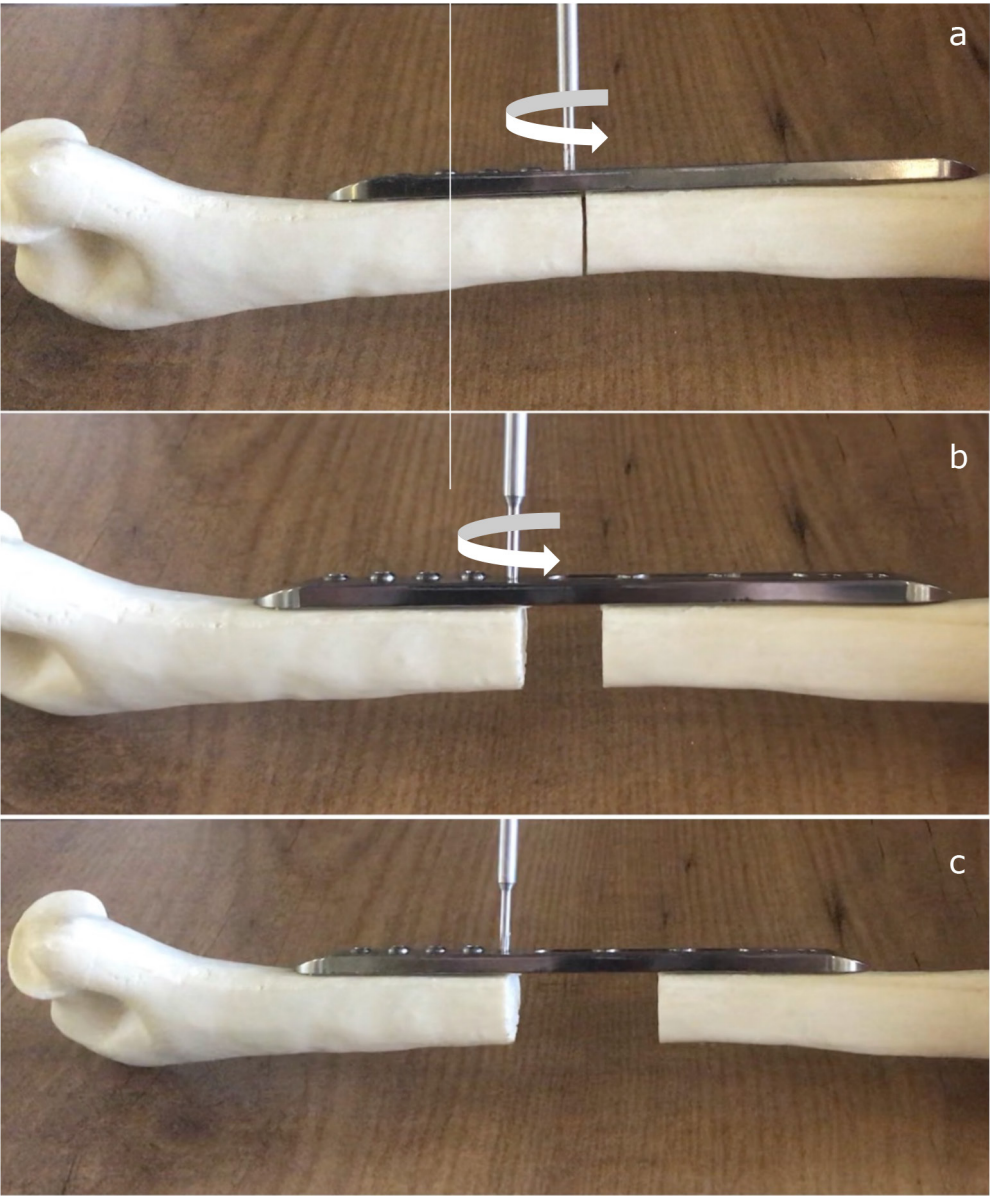
Bone lengthening technique up to 4 cm. (a) Fixation of the plate to bone surface and rotating the screwdriver on white arrow direction in order to initiate lengthening, (b) Keep turning the pinion mechanism to achieve bone lengthening, (c) End of the bone lengthening.

**Figure 4:**
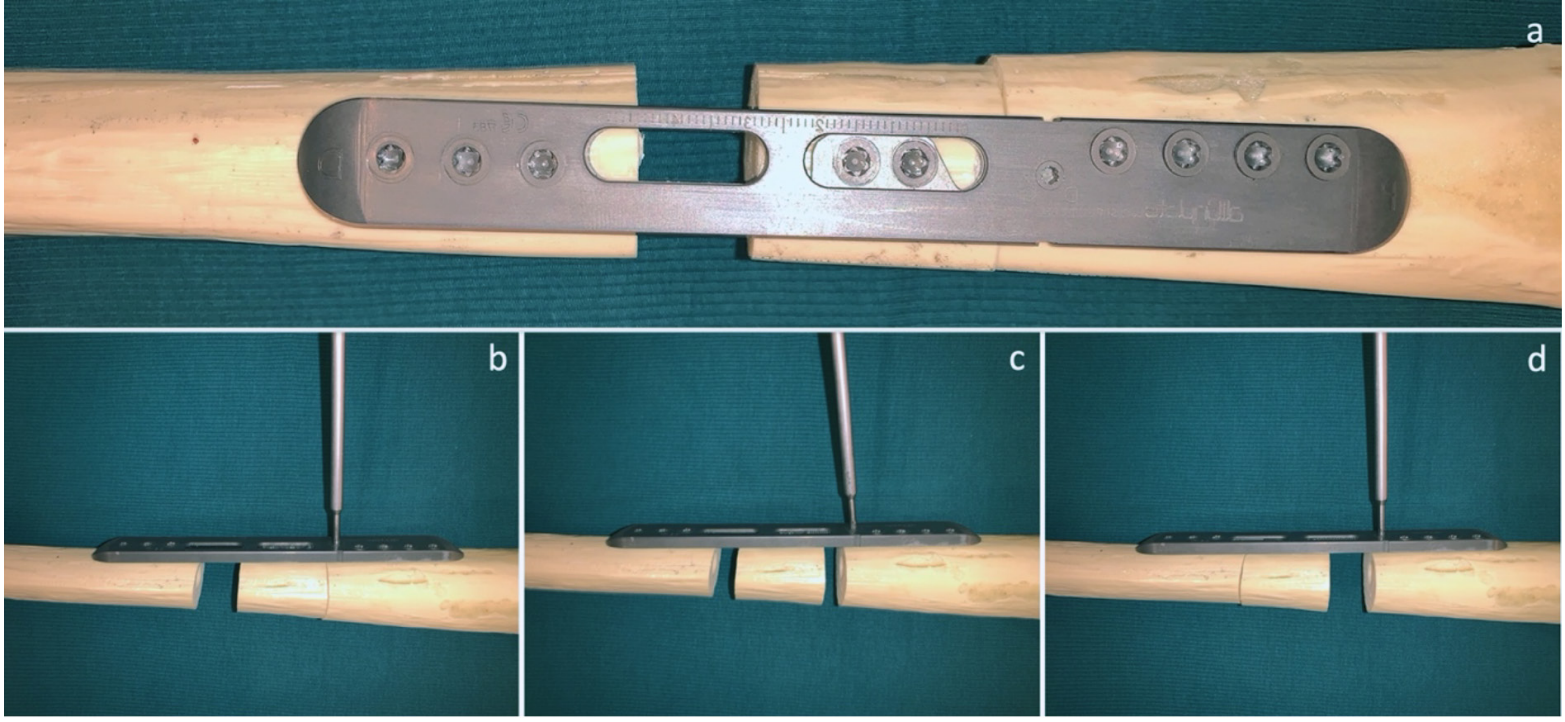
Fixation of ABP on sawbone with 15 mm bone defect model. (a) Top view, (b) Initial-stage of bone segment transfer, (c) Mid-stage of bone segment transfer, (d) End-stage of bone segment transfer.

Surgically, ABP must be placed parallel to the long axis of the bone. The dynamic part must be placed distal to the osteotomy site. Initially, static part is fixed to the bone with its proximal screws then the dynamic part is fixed with its locking screws to the bone. Subsequently, osteotomy is performed between the closest screws in the static and dynamic part or fracture site is adjusted to be placed this region. Application of the ABP with this method allows both compression and distraction in the osteotomy or fracture site by rotating the pinion mechanism screw with a screwdriver (Figure 3). Furthermore, the new plate design allows surgeons to transfer the bone segment in the treatment of bone defects. Similar steps as mentioned above with a second osteotomy is enough to maintain segment transfer (Figure 4)

## 4. Discussion

ABP brings advantages of intramedullary nails and external fixators in segmental bone transfers as well as reduces their potential complications. It solves the main problem, pin tract infection of the Ilizarov technique which was observed around 10%–20% [7]. It is a self-internal splint which do not require a secondary implant or procedure until consolidation is achieved. It could be applied by minimally invasive technique which protects the vascularization of bone and enhance healing [17]. ABP is a user-friendly device that protects the axial alignment of the bone during distraction. We believe that there is a gap in the literature in the optimal implant for the upper extremity distraction osteogenesis and paediatric bone lengthening [18–20]. ABP can fill this gap in upper extremity with its extramedullary design and eliminating the neurovascular complications of external fixators. In paediatric population, ABP can be used safely without intervening the growth plates. 
